# Acute Pericarditis After Use of Electronic Cigarettes: A Case Report

**DOI:** 10.7759/cureus.49810

**Published:** 2023-12-01

**Authors:** Minh Tran Duc, Yen Nguyen, Duc Nguyen Hung, Lam Truong Hoai, Phong Nguyen Xuan

**Affiliations:** 1 Caridiology, Tam Anh Hospital, Hanoi, VNM; 2 Cardiology, Tam Anh Hospital, Hanoi, VNM; 3 Radiology, Tam Anh Hospital, Hanoi, VNM

**Keywords:** vaping, vapes, e-cigarette, electronic cigarette, : acute pericarditis

## Abstract

Acute pericarditis is the most common pericardial disease in clinical practice and frequently in young and middle-aged people. The past decade has dramatically increased electronic cigarettes or vapes in developing countries. However, there are no case reports describing vaping-induced acute pericarditis. This report describes a case of a 27-year-old male who presented with acute onset chest pain after using an electronic cigarette. His ECG showed typical pericarditis with diffuse ST-segment elevation and downsloping TP segment. The patient responded to the medical therapies of non-steroidal anti-inflammatory drugs (NSAIDs) and colchicine, but serum troponin T went up. In this case report, the authors have shared their opinions on how to handle this situation.

## Introduction

In clinical practice, acute pericarditis is encountered most frequently among pericardial diseases. It is worth mentioning that pericarditis is diagnosed in around 5% of patients admitted to emergency for chest pain unrelated to acute myocardial infarction [1]. Chest pain in acute pericarditis is mainly pleuritic and worsens in the supine position with deep inspiration. The differential diagnosis is somewhat challenging due to ST-segment elevation caused by certain ECG changes and an increase in troponin T level [2]. However, diffusive concave ST-segment elevation is a typical ECG finding in acute pericarditis, accompanied by PR depression. Acute pericarditis can commonly be caused by both infectious and non-infectious factors. Viral infections are the most common cause of pericarditis in developed countries, while tuberculosis is the predominant cause in developing countries [3]. The use of electronic cigarettes or vaping has seen a significant increase, which could potentially be a new cause of pericarditis. Here, we present a case of a young male who developed acute pericarditis suspected to be caused by vaping.

## Case presentation

A 27-year-old male presented to the emergency department with chest pain that lasted for two days without any medical history. His pain was substernal and radiating to the left shoulder. It was augmented with cough or deep inspiration. He did not report any recent respiratory symptoms or any antecedent viral symptoms. He revealed that he used an electronic cigarette two days before the onset of chest pain. It was his first time using an e-cigarette.

**Figure 1:**
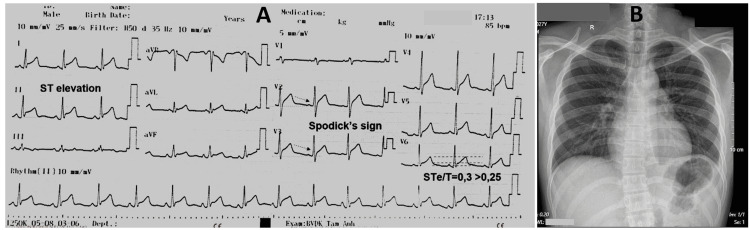
Admission ECG and chest X-ray on day 1

On admission to the emergency department, his temperature was 36.7°C, blood pressure was 123/78 mmHg, heart rate was 85 beats/minute, respiratory rate was 17 breaths/minute and regular pulse oximetry was 97% on room air. Physical examination showed no pericardial friction rub and normal heart sounds. The chest was not tender and the sounds of breath were normal. The initial 12-lead electrocardiogram (Figure 1A) revealed sinus rhythm, a rate of 85 beats per minute with concave and diffuse ST-segment elevation in leads I, II, aVL, aVF, and V2 through V6. There was depression only in the ST segment in aVR. The PR segment did not have any significant differences from isoelectric in any lead. Lead II, V2 through V5 showed a downward-sloping TP segment.

The patient’s chest X-ray confirmed a clear lung with no abnormality (Figure 1B). The transthoracic echocardiogram (TTE) revealed that his cardiac structure and function were normal without any pericardial effusion. The initial laboratory workup is shown in Table 1.

**Table 1:** Initial laboratory workup on admission NT-proBNP: N-terminal pro b-type natriuretic peptide; COVID-19: coronavirus disease 2019; HbsAg: hepatitis B surface antigen; HCV: hepatitis C virus *Manufacturer details: QIAGEN, Hilden, Germany

Test	Results	Reference ranger
White blood cell count (G/L)	15.57	4.0-10
C-reactive protein (mg/dL)	10.7	0.0-0.5
Troponin T on admission (pg/mL)	4.25	0.0-14.0
Troponin T at 3 hours	5.11	
NT-proBNP (pmol/L)	5.17	0.0 – 14.0
COVID-19 Ag rapid test	negative	negative
Tuberculosis (QuantiFERON-TB Gold Plus*)	negative	negative
HbsAg rapid test	negative	negative
Anti-HCV rapid test	negative	negative
Anti=HIV rapid test	negative	negative
Influenza virus A&B	negative	negative

After analyzing laboratory findings, the patient’s medical history, and previous hospital admission data, it was believed that recurrent pericarditis was present. He was admitted to the hospital for additional treatment and clinical investigations.

**Figure 2:**
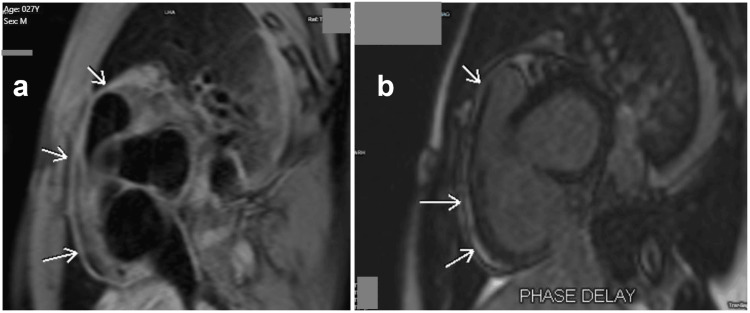
(a) T2-weighted spin echo image depicting the acutely inflamed pericardium which appears high signal (arrow); (b) Late gadolinium enhancement sequence revealing avid uptake of contrast by the inflamed pericardium (arrow)

He was started on oral high-dose with aspirin 325 mg every eight hours, colchicine 0.5 mg, and esomeprazole 40 mg daily. With the prescribed treatment, the patient's condition improved, the chest pain resolved completely within three days and inflammation markers began to diminish: white blood cell count 15.57→6.4 G/L, elevated C-reactive protein 10.7→1.68 mg/dL. Despite this, the serum troponin T was repeated at 303 pg/mL and there were no changes in the ECG and TTE. The presence of pericarditis without significant myocardial injury was further demonstrated by cardiovascular magnetic resonance (CMR). A high signal was seen in the T2-weighted spin-echo image of the acutely inflamed pericardium (Figure 2a). Late gadolinium enhancement (LGE) sequence revealed an avid uptake of contrast by the inflamed pericardium (Figure 2b). No evidence of pericardial effusion was found after performing CMR.

Consequently, he was advised to keep taking aspirin and colchicine. He was counseled regarding the possibility that his vaping was implicated in the etiology of the pericarditis. He was discharged on day 4 and instructed to follow up in the cardiology clinic.

## Discussion

Acute pericarditis is diagnosed based on two of the following criteria: chest pain, pericardial rub, new widespread saddle-shaped ST-segment elevation and/or PR segment depression, and non-trivial new or worsening pericardial effusion [3]. Our patient experienced chest pain and had a diffuse ST-segment elevation on his ECG. Moreover, we would like to draw the reader’s attention to interesting ECG findings: Spodick’s sign and the ST/T ratio. Spodick’s sign is a downsloping TP segment in about 80% of patients affected with acute pericarditis [4]. In this case, ST elevation to T wave amplitude was 0.3 in lead I, V5, and V6. In an earlier intriguing study, the authors concluded that a ratio of the ST amplitude segment to the amplitude of the T-wave ≥0.25, in leads I, V4, V5, and V6, was predictive of acute pericarditis [5].

ECG findings and negative troponin T levels ruled out the possibility of acute myocardial infarction. Although this was the case, the serum troponin T was repeated at 303 pg/mL and there were no modifications in the ECG. CMR confirmed acute pericarditis and excluded concomitant myocarditis as well as other differentials. Also, there was no evidence of pericardial effusion In the T2-weighted spin-echo image of the acutely inflamed pericardium, a high signal was observed and late gadolinium enhancement focused pericardium around the right ventricle. The severity of inflammation may be related to baseline LGE observed on CMR [6]. Regrettably, this approach did not identify the cause of pericarditis, but it did not necessitate coronary angiography to rule out coronary artery disease.

In developing countries, there has been a significant increase in the use of electronic cigarettes or vaping over the past decade. In Southeast Asia, the current prevalence of electronic cigarette use is between 3.3% and 11.8% [7]. Although there are still many unknowns about the negative effects of vaping, there have been reports of an electronic cigarette or vaping-associated lung injury in users thus far [8]. Clinicians need to be informed about the potential cardiovascular complications of vaping, such as pericarditis. Our patient mentioned using an electronic cigarette two days before experiencing chest pain. He had never used electronic cigarettes before. We don't know exactly what ingredients were in the electronic cigarette he used. In our opinion, electronic cigarette smoking has the potential to cause acute pericarditis.

His symptoms improved quickly to the conventional treatments of high-dose aspirin, colchicine, and cessation of vaping. Insufficient treatment of pericarditis can be detrimental and lead to irreversible complications, such as myopericarditis. An interesting study demonstrated that non-steroidal anti-inflammatory drugs (NSAIDs) are safe in patients with myopericarditis [9]. Therefore, he was advised to continue taking aspirin and colchicine. 

## Conclusions

There are still numerous unanswered questions regarding the potential cardiovascular complications associated with vaping. It is important to note that no cases of acute pericarditis caused by vaping have been reported in the literature. We presented the case of a young male who was suspected of acute pericarditis as a result of vaping. The ECG revealed a typical pattern of pericarditis, characterized by diffuse ST-segment elevation and a downward-sloping TP segment. Despite the administration of NSAIDs and colchicine, the patient's serum troponin T levels increased. In this report, we shared our insights on how to approach this situation. Our case report emphasized the significance of obtaining an accurate medical history, analyzing the ECG, and utilizing CMR for the diagnosis of pericarditis. We are hopeful that our description of this case will prove valuable to others.
